# Genetically-Informed Patient Selection for iPSC Studies of Complex Diseases May Aid in Reducing Cellular Heterogeneity

**DOI:** 10.3389/fncel.2017.00164

**Published:** 2017-06-13

**Authors:** Stephanie D. Hoekstra, Sven Stringer, Vivi M. Heine, Danielle Posthuma

**Affiliations:** ^1^Department of Complex Trait Genetics, Center for Neurogenomics and Cognitive Research, Vrije Universiteit AmsterdamAmsterdam, Netherlands; ^2^Department Pediatrics/Child Neurology, VU University Medical CenterAmsterdam, Netherlands; ^3^Department of Clinical Genetics, VU University Medical Center, Amsterdam NeuroscienceAmsterdam, Netherlands

**Keywords:** IPSC, schizophrenia, variability, disease models, genetics, psychiatric diseases, statistical power, stem cells

## Abstract

Induced pluripotent stem cell (iPSC) technology is more and more used for the study of genetically complex human disease but is challenged by variability, sample size and polygenicity. We discuss studies involving iPSC-derived neurons from patients with Schizophrenia (SCZ), to exemplify that heterogeneity in sampling strategy complicate the detection of disease mechanisms. We offer a solution to controlling variability within and between iPSC studies by using specific patient selection strategies.

## Introduction

Induced pluripotent stem cells (iPSCs) are increasingly being used to investigate disease mechanisms underlying complex diseases, like schizophrenia (SCZ), autism spectrum disorders and major depressive disorder. The genetic architecture of complex diseases is characterized by its polygenic nature, with thousands of genetic loci increasing disease risk, and by various combinations of risk loci carried by different patients. Such genetic heterogeneity may have undesirable effects on the outcomes and the interpretations of iPSC studies. When genetic heterogeneity is not controlled and participants in iPSC studies are e.g., selected based on the presence or absence of a polygenic disease, the cases may have partly or even completely different risk alleles that contribute to the disease. Especially since iPSC studies typically involve few participants (<30), an unlucky draw of cases (yet the same holds for controls) may result in genetically heterogeneous cases (and controls). If such genetic heterogeneity is related to heterogeneity at the cellular level, variability at a biological read-out will increase, which will in turn decrease the statistical power to detect a difference in the biological read-out between cases and controls. Here we will discuss the importance of addressing genetic heterogeneity and patient selection strategies in the design of iPSC studies for complex disorders.

## Heterogeneity and statistical power

When genetic heterogeneity is not controlled, differences in biological read-out seen between cases and controls in study 1 may be not be found in study 2. This can reflect a false positive finding in study 1, but may also reflect genetic heterogeneity between studies. This is unfortunate, as replication is important and will solidify the conclusions of a study.

**Figure 1:**
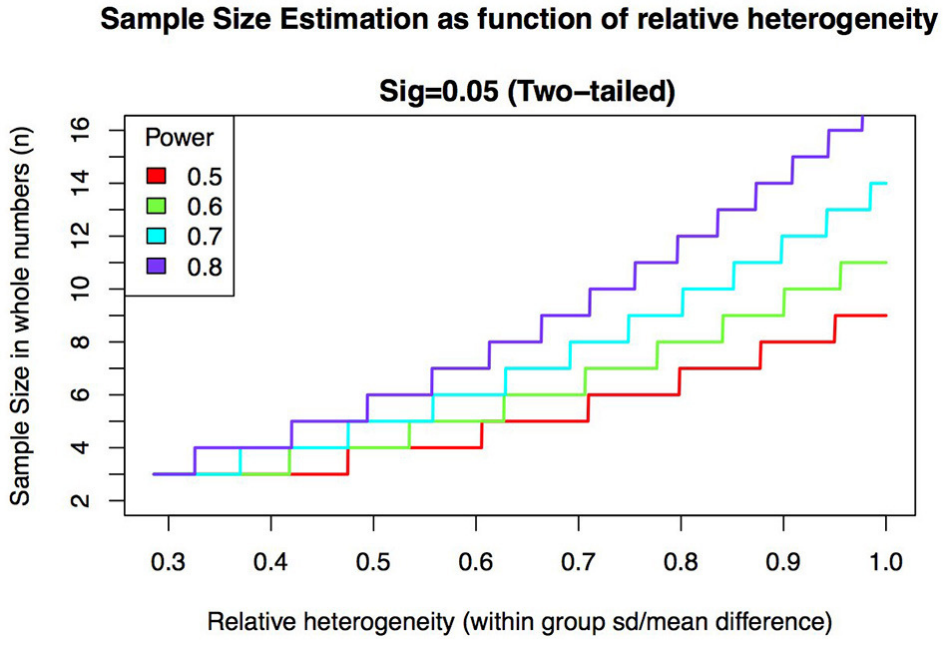
Required sample size increases as a function of relative heterogeneity for different levels of statistical power. Relative heterogeneity is defined here as the ratio between within-group standard deviation and mean group difference.

To illustrate how the polygenic background of complex disorders affects the statistical power of iPSC studies, we calculated the effect of variability (induced by genetic heterogeneity) in the biological readout on the power to detect statistically significant differences in the readout between cases and controls (Figure 1). The results presented in Figure 1 are based on a power analysis in which we assume a design with two contrast groups (e.g., case vs. control) and a continuous outcome measure (e.g., expression of proteins of interest). Heterogeneity between cells of different subjects within each group is expressed in standard deviations (sd). Without loss of generality we define the relative heterogeneity as the ratio between the within-group standard deviation and the mean difference between the groups. If the mean difference is 1, this measure of heterogeneity is simply the standard deviation in outcome within each group. Figure 1 shows how large the variability within a group is relative to the observed mean difference between the groups. Thus the larger this heterogeneity, the larger the required sample size becomes. Ideally the variability within each group is much smaller than the variability between the groups. On the other hand, when the relative heterogeneity is large, say 1.2, the standard deviation is 20% points larger than that of the observed average group difference. In this case it would be difficult to detect a significant difference between groups. Figure 1 shows that with samples sizes around 5 the optimal ratio is 0.5. However, since we cannot control effect sizes of the biological read-out (i.e., the difference in the measured cellular phenotype between cases and controls), it would be advisable to reduce variability by reducing genetic heterogeneity within one group.

One way to increase statistical power is to increase sample size. This would make unlucky draws less likely. However, due to the current labor-intensive nature of iPSC studies, sample sizes above 10–30 individuals are often not feasible, and alternative strategies are needed. One such strategy is to use genetically-informed decisions in patient (and control) selection. By selecting genetically homogeneous cases and controls, within-group variance can be reduced, which is a critical determinant in both increasing statistical power and evaluating results from iPSC studies for complex disease (Figure 1).

## Schizophrenia

To illustrate the importance of reducing genetic heterogeneity we discuss several examples in the context of (SCZ), a complex disorder (SchizophreniaWorking Group of the Psychiatric Genomics Consortium et al., 2015) for which already a number of iPSC-based studies have been published and in which a number of different patient selection strategies have been applied.

Table 1 lists current iPSC studies investigating SCZ with their selection of patients and controls. To illustrate differences in patient selection between studies we also list Odds Ratios (ORs) and penetrance for SCZ and other disorders. As shown in this table, several studies selected cases for the presence of a specific SCZ-associated genetic component of large effect. These studies selected specific genetic variants to reduce genetic and possibly cellular heterogeneity, rather than selecting on diagnosis. Wen et al. (2014), Pak et al. (2015), and Siegert et al. (2015) investigated a single variant in DISC1, NRXN1, and mir137, respectively (also see Table 1). Each of these three studies reported presynaptic deficits in carriers vs. non-carriers, suggesting that these presynaptic deficits are important in the etiology of SCZ. Specifically all three implicated an important role for the release probability of vesicles. The deficit in vesicle release reported by these studies caused a decrease in spontaneous mini excitatory postsynaptic currents (mEPSC) and EPSC amplitude. However, the difference in EPSC amplitude in one study depended on the control taken for comparison (Wen et al., 2014).

**Table 1:** Summar of iPSC research to SCZ.

**References**	**Genetically Informed**	**Patients**	**Controls**	**OR**	**Penetrance**	**Penetrance OTHER**	**Generalization of data**	**Confirmation**	**SCZ Phenotype**	**Other Phenotype**	**Potential of bias**
Brennand et al., 2011	No	Male early onset SCZ (suicide), brother (SCZ) and sister (SAD), unrelated patient	One commercially availabe new born control (male), 2 males (20–22 y/o) and 4 females (22–25 y/o)	Unknown	Unknown	Unknown	Good	No	Low	Low	No
Paulsen et al., 2012	No	Clozapine resistant female patient	H09 (female) line, male control (commercially available fibroblasts)	Unknown	Unknown	Unknown	Good	No	Low	Low	No
Robicsek et al., 2013	No	Male with affected mother, female with affected mother, male with affected father and grandfather, all responding to clozapine	2 controls	Unknown	Unknown	Unknown	Good	No	Low	Low	No
Brennand et al., 2015	No	Patients from Brennand et al., 2011	One commercially available new born control, 1 other male (20 y/o) and 4 females (22-25 y/o) all from Brennand et al., 2011	Unknown	Unknown	Unknown	Good	No	Low	Low	No
Yu et al., 2014	No	Patients from Brennand et al., 2011	4 controls from Brennand et al., 2011	Unknown	Unknown	Unknown	Good	No	Low	Low	No
Hook et al., 2014	No	Male early onset SCZ and two males from Brennand et al., 2011	Newborn fibroblasts, 22 y/o female, 25 y/o female	Unknown	Unknown	Unknown	Good	No	Low	Low	No
Paulsen et al., 2014	No	Patients from Paulsen et al., 2012	Controls from Paulsen et al., 2012	Unknown	Unknown	Unknown	Good	No	Low	Low	No
Hashimoto-Torii et al., 2014	No	Patients from Brennand et al., 2011	5 controls from Brennand et al., 2011	Unknown	Unknown	Unknown	Good	No	Low	Low	No
Wen et al., 2014	Yes	Father (MD) and daughter (SCZ), both DISC1 frameshif	Mother/wife of patients, daughter/sister of patients, one unrelated male control	LOD 3.6^***^	Unknown	LOD 7.2^***^	Low	Yes	High	High	Yes
Yoon et al., 2014	Yes	3 SCZ patients carrying a 15q11.2 deletion	5 family members	2.15^**^	2%^*^	11%^*^	Low	Yes	High	High	Yes
Bundo et al., 2014	Yes	2 patients with 22q11.2 deletion (both female)	16 y/o female	NA^**^	12%^*^	88%^*^	Low	Yes	High	High	Yes
Topol et al., 2015	No	Patients form Brennand et al., 2011	6 controls from Brennand et al., 2011	Unknown	Unknown	Unknown	Good	No	Low	Low	No
Murai et al., 2016	Yes	Patients and controls from Wen et al., 2014	Mother/wife of patients, daughter/sister of patients, one unrelated male control	LOD 3.6^***^	Unknown	LOD 7.2^***^	Low	Yes	High	High	Yes
Pak et al., 2015	Yes	Induced NRXN1 deletion and truncation in H01	H01	9.01^**^	6.4%^*^	26%^*^	Low	Yes	High	High	Yes
Narla et al., 2017	No	Patients from Brennand et al., 2011	One commercially available new born control, 1 other male (20 y/o) and 2 females (20-25 y/o) all from Brennand et al., 2011	Unknown	Unknown	Unknown	Good	No	Low	Low	No
Topol et al., 2016	No/yes	Patients from Brennand et al., 2011; 10 additional patients, 6 carrying several CNVs	One commercially availabe new born control, 1 other male (20 y/o) and 4 females (22-25 y/o) from Brennand et al., 2011, 10 additional controls (5 males, 5 females)	Variety	Variety	Variety	Very good	Yes	High/Low	Low	No
Marcatili et al., 2016	No	Clozapine responsive patient	Commercially available control	Unknown	Unknown	Unknown	Good	No	Low	Low	No
Marsoner et al., 2016	No	Clozapine responsive patient	None	Unknown	Unknown	Unknown	Good	No	Low	Low	No
Siegert et al., 2015	Yes	Carriers of SNP in mir-137	Two subjects not carrying the risk SNP in mir137	Variety	Unknown	Unknown	Low	Yes	Low	High	Yes
Toyoshima et al., 2016	Yes	Patients from Bundo et al., 2014	36 y/o female	NA^**^	12%^*^	88%^*^	Low	Yes	High	High	Yes
Lin et al., 2014	Yes	8 unrelated patients with 22q11.2 deletion (SCZ or SAD)	7 controls	NA^**^	12%^*^	88%^*^	Low	Yes	High	High	Yes

*Table contains information about patient and control selection, the odds ratio (OR) for SCZ of the chosen genetic variant (if known), the penetrance for SCZ and for other disorders*.

*The table also compares the ability of generalization of results, confirmation if the genetic variant chosen can be verified by gene editing, expected effect size for SCZ, expected effect size for other disorders, bias toward synaptic genes. SAD, schizoaffective disorder*,

* *adapted from Rees et al. (2014)*,

** *adapted from Kirov et al. (2014)*,

*** *adapted from Blackwood et al. (2001) (translocation rather than deletion since numbers for deletion were unavailable)*.

Another iPSC study selected SCZ patients with a high likelihood of a genetic burden based on family history rather than carriers of a specific variant, vs. controls that did not have a familial burden. In this study no such presynaptic deficits were reported (Brennand et al., 2011). Comparing these outcomes to the previously mentioned three studies may cast doubt on the presynaptic involvement in SCZ. However, the results from these different studies cannot be directly compared as SCZ is a polygenic disorder and the studies (implicitly) selected patients carrying a variety of risk variants. Different risk factors might affect different cellular pathways that do not lead to presynaptic deficits. Interestingly, Yu et al. (2014), who studied the same patients as Brennand et al. (2011) but only a used a subset of the controls used by the same group (Table 1), did find a decrease in mEPSCs frequency and amplitude as reported by the Wen et al. (2014), Pak et al. (2015) and Siegert et al. (2015). The latter underlines the impact of specific patient—control combinations. This idea is further supported by the findings of Wen et al. (2014), who reported that differences in EPSC amplitude were dependent on the chosen control (related or unrelated). Presynaptic deficits might thus still be a causal mechanism in SCZ. The important issue here is that comparison of results between studies assumes that the same contrast groups were used, while this may not always be the case. In addition we would like to point out another important issue; in the studies where individuals carried the selected genetic variants, not all individuals were known to be diagnosed with SCZ (Wen et al., 2014; Siegert et al., 2015). If the cells are derived from carriers who do not have the disease and that are passed the age of onset, no definite conclusions can be drawn about the causal role of the reported presynaptic deficits in SCZ, because clearly the presence of the genetic variant as well as the presynaptic deficits do not co-occur with SCZ disease status in all individuals. Causal inferences in the context of SCZ in these studies would have been more reliable when all carriers would have been diagnosed with SCZ. Another important issue is that there seems to be a bias toward variants known to be affecting the synapse. Although SCZ has been hypothesized to be a disease of the synapse, there are studies showing other pathways may have a big impact in SCZ, such as GFAP overexpression and oxidative stress (Paulsen et al., 2012; Robicsek et al., 2013; Toyoshima et al., 2016).

As described above, causality is difficult to claim in complex trait diseases. These studies exemplify that results are (i) highly dependent on the selection of subjects (ii) hard to interpret due to a lack of fully penetrant and disease specific variants. This calls for a more genetically informed selection of patients and controls, to control for genetic background, to improve comparison between studies and to investigate causality.

## Using specific variants with large effect

Researchers often choose to investigate a specific variant with large effect (mostly CNVs). An important issue when focusing on a single CNV is that many CNVs that have been associated with SCZ have also been associated with other psychiatric disorders such as major depressive disorder or autism (Kirov et al., 2014; Wen et al., 2014; and also mentioned by Pak et al., 2015) and this may occur in the same family. This makes it less likely that these CNVs are SCZ-specific and thus impedes inferences on the specificity of detected cellular phenotypes for SCZ. Thus, both genetic heterogeneity (e.g., people carrying the same rare genetic variant but of completely different polygenic risk) and pleiotropic genetic effects (i.e., the same genetic variant causes multiple diseases) complicate the detection of robust cellular phenotypes that are causally linked to the targeted disorder. Revealing common pathways causal to psychiatric diseases is of great value, and should be investigated further. However, claims of specificity to one particular disease are incorrect when based solely on research to nonspecific genetic background such as CNVs in complex trait genetics. Despite the fact that the role of such variants in other disorders can also be of interest, revealing specific pathways to specific disorders might lead to development of more targeted drugs for specific phenotypes with very little side effects. Furthermore, the fundamental knowledge of why some individuals carrying the same genetic variant develop SCZ and others develop major depressive disorder will enlighten biological processes as well as genetic ones.

Although selecting for a rare genetic variant of large effect reduces genetic heterogeneity and increases statistical power, rare variants carriers also have a significant predisposition for SCZ caused by common variants (Tansey et al., 2016). This complicates interpretation of results arising from gene editing studies are truly causal to a specific disease. One such example is the study of functional implications of the NRXN1-gene variants (e.g., Pak et al., 2015). NRXN1 is included in a CNV that is one of the most replicated findings for SCZ with Odds Ratio's (ORs) in the order of 9.01 (Kirov et al., 2014). The penetrance of this CNV for SCZ is, however, only 6.4% (Kirov et al., 2014), which means that other causal factors are needed to induce SCZ. Therefore, experimental studies focusing on NRXN1 risk carriers alone may not hold the key to understanding SCZ. The general idea is that patients tend to carry many common risk alleles as well on top of the rare variant associated with SCZ (Tansey et al., 2016). Thus, studies based on gene editing without controlling for genetic background are highly suitable for investigating gene function. However, focusing on a single variant may not always provide sufficient information on cellular pathways involved in SCZ. The use of gene editing could be advantageous when used in combination with high polygenic risk score lines. By introducing a SCZ-associated CNV in lines with high polygenic risk scores an enhanced SCZ phenotype is expected (while in lines with low polygenic risk scores no SCZ phenotypes are expected due to small penetrance).

To claim causality, it is important to include knowledge on the background regarding polygenic risk when selecting patients. The use of polygenic risk scores, especially in combination with the gene editing gives the opportunity of creating continuous variable for risk. This allows correlational analysis between risk and phenotype. If the phenotype correlates with risk score, the probability of a false positive will be very low.

## Decreasing heterogeneity by selecting homogeneous cases and controls

**Figure 2:**
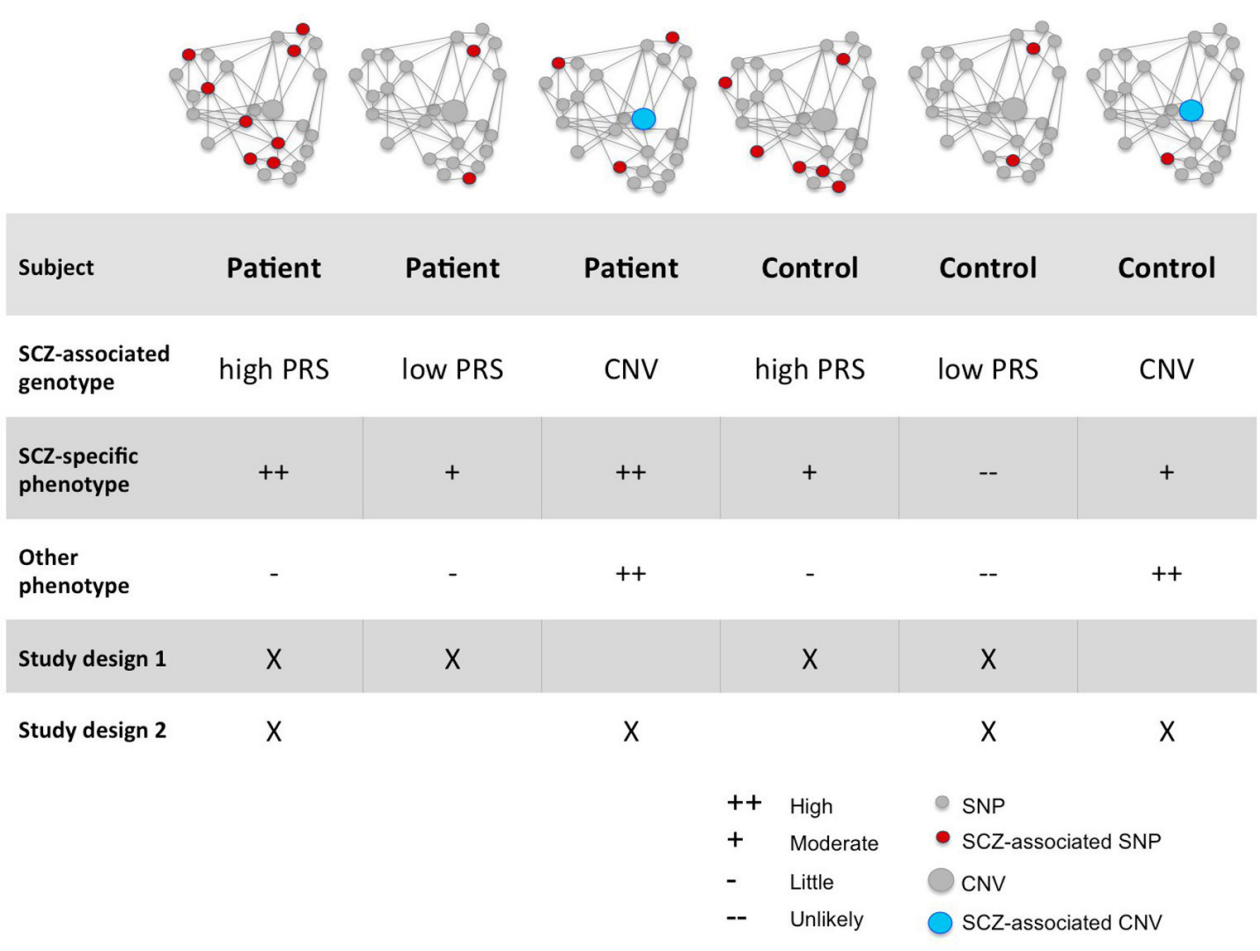
Proposed participant selection strategies. This figure illustrates all types of subjects: subjects with high and low burden carrying only common or also rare variants. Below the schematic representation of each subject one can find a comparison for the two factors: the chance of finding a SCZ associated phenotype and the chance of finding a phenotype associated with other disorders. Two ideal strategies (discussed in the main text) are also illustrated.

Studies aiming to reveal causal biological pathways for complex diseases will benefit from improved strategic patient selection, to control for the effects of genetic heterogeneity and pleiotropy. We propose two improvements in patient selection aimed at increasing genetic homogeneity as well as effect sizes: (i) select patients carrying a specific disease-associated genetic variant with a high penetrance and large effect size, or (ii) select patients with high polygenic risk based on common genetic variants. For both strategies the ideal design would be to include four groups of individuals: patients with and without the disease penetrant variant/high polygenic risk and controls with and without the disease penetrant variant variant/high polygenic risk (Figure 2). This four-way study design allows drawing conclusions on the validity of detected cellular differences for the disease. For example, if suboptimal function of a cellular phenotype is causally related to a disease, the largest phenotype is expected to be most affected in patients with the penetrant variant or high PRS, then in patients without the penetrant variant or low PRS, then in controls with a penetrant variant or high PRS, and lastly in controls without the penetrant variant or low PRS. As described above, many current study designs only include patients carrying known rare variants (with varying penetrance) and controls without the variant, or they choose to include patients and controls solely based on diagnosis. This limits our ability of linking detected phenotypes to the targeted disease. Topol et al. (2016) chose an approach (Table 1) similar to the approach proposed here, i.e., combining patients based on diagnosis only (no SCZ associated CNVs) and patients carrying a variety of SCZ associated CNVs. This design allows the finding of a general phenotype present in both groups of patients. As mentioned before, controlling genetic background by investigating polygenic risk scores allows correlation of the phenotype to genetic burden, reducing the number of potential (no specific) phenotypes and thus decreasing the chance of false positives.

## Decreasing heterogeneity by studying families

Another way of controlling for genetic heterogeneity by using genetically-informed selection strategies is by using family members, which offers a natural way of matching for genetic background. Family studies have been crucial for understanding the pathology of SCZ, as they offer a model with relatively low genetic variance and therefore high power. Most families affected with SCZ carry a rare variant, with relatively high OR's and relatively large cellular effects as compared to common variants. Due to the genetic relationship any background effects are also partially matched. This is illustrated by the EPSC amplitude reported by Wen et al. (2014) as discussed above. Although rare variants explain only a small percentage of the general SCZ cases, they can be helpful in unraveling cellular pathways involved in SCZ. The presence of a single, relatively large variant with a relatively high penetrance facilitates rescuing of observed phenotypic consequences by gene-editing. However pleiotropic genetic effects may still complicate the interpretation of results and researchers should investigate the presence of other (common) variants present in patients and controls. As seen in Wen et al. (2014) family members carrying the CNV of interest can develop SCZ while other members develop another disease such as major depressive disorder. This is likely the effect of common variants carried by each individual besides the CNV. Also in this setting research will benefit from controlling for genetic background and from reporting on common variants carried by the cases and controls.

In summary, selecting a genetic variant with high penetrance directly circumvents patient heterogeneity as a confounding factor. If a variant is chosen for its high penetrance and its large effects on risk for the targeted diseases, the effects on a biological phenotype can be expected to be large, thereby increasing detectability and statistical power. In practice however, choosing a single variant may not be straightforward; highly penetrant variants may not (yet) be known for a disease (Falk et al., 2016), or they may be related to other diseases as well (Kirov et al., 2014). If no good genetic candidates are available, the second strategy provides a good alternative. In this selection design, patients and controls are selected with high and low polygenic burden; this strategy is in line with the general assumption that many common variants of small effect converge on a biological pathway or function; i.e., heterogeneity may exist at the level of alleles or affected genes but will be less at the level of biological pathways. This strategy is therefore expected to enhance effect sizes as it involves selection on the accumulated effect of multiple risks. Reducing genetic heterogeneity will increase the statistical power of studies and will help researchers to overcome a great issue in the stem cell field: sample sizes. As shown in Figure 1, needed sample sizes (assuming 1 iPSC clone per individual) depend on mean differences between groups (effect size) and on the variance within the groups. The use of extremely different cases and controls will help increasing the mean difference between patients and controls, and by ensuring all cases and all controls are genetically matched, genetic heterogeneity within groups will be reduced. This will then lead to an increase in statistical power with smaller sample sizes.

## Conclusion

The decrease in (genetic and phenotypic) heterogeneity will reduce the number of (nonspecific) phenotypes we observe within and between studies and therefore will increase the chance of finding SCZ-associated causal pathways. In addition, targeted participant selection facilitates comparing results across different studies for replication purposes. As iPSC research is already challenged by variability (Falk et al., 2016), stratification of patient selection as described above to improve statistical power and comparison between studies will therefore be of utmost importance.

## Author contributions

All authors listed, have made substantial, direct and intellectual contribution to the work, and approved it for publication. VMH and DP concaived the study. SDH, VMH, and DP wrote the manuscript, SDH conducted the literature research. SS coducted the statistical power simulations.

### Conflict of interest statement

The authors declare that the research was conducted in the absence of any commercial or financial relationships that could be construed as a potential conflict of interest.
